# Magnetic mineral characteristics, trace metals, and REE geochemistry of river sediments that serve as inlets to Lake Limboto, Sulawesi, Indonesia

**DOI:** 10.1016/j.dib.2019.104348

**Published:** 2019-08-22

**Authors:** Satria Bijaksana, Raghel Yunginger, Abd Hafidz, Mariyanto Mariyanto

**Affiliations:** a Faculty of Mining and Petroleum Engineering, Institute Teknologi Bandung, Bandung 40132, Indonesia; b Department of Physics, Faculty of Mathematics and Natural Sciences, Universitas Negeri Gorontalo, Gorontalo 96128, Indonesia; c Department of Geophysical Engineering, Faculty of Civil, Environmental and Geo Engineering, Institute Teknologi Sepuluh Nopember, Surabaya 60111, Indonesia

**Keywords:** Lake limboto, Magnetic properties, Trace metals, REE, River sediments

## Abstract

This article presents magnetic mineral characteristics, trace metals, and REE geochemistry of river sediments that serve as inlets to Lake Limboto, Sulawesi, Indonesia related to article entitled “Lithogenic and anthropogenic components in surface sediments from Lake Limboto as shown by magnetic mineral characteristics, trace metals, and REE geochemistry” [1]. River sediments were obtained from three rivers, namely Alopohu, Bionga, and Talumelito. Sieved sediments were subjected to magnetic susceptibility measurements as well as geochemical analyses that include AAS analyses for trace metals and ICP-OES for REE. Extracted magnetic grains were also subjected to magnetic hysteresis analyses as well as XRD and SEM analyses. These data are invaluable in identifying the contribution of each river (and its catchment area) to the surface sediments of Lake Limboto.

**Table:** Specifications Table

Subject area	Geophysics
More specific subject area	Environmental magnetism
Type of data	Tables, Graphs, Figures
How data was acquired	1. Bartington MS2 susceptibility (equipped with dual-frequencies MS2B sensor) made by Bartington Instrument Ltd., Oxford, UK was used to measure magnetic susceptibility of bulk samples. 2. Oxford Instrument 1.2H/CT/HT vibration sample magnetometer (VSM) made by Oxford Instrument, Oxfordshire, UK was used to measure the hysteresis parameters of magnetic grains. 3 Rigaku SmartLab X-Ray Diffractometer made by Rigaku Corp., Tokyo, Japan was used for mineral identification of magnetic grains. 4. JEO JSM-6510A scanning electron microscope (SEM) made by JEOL Ltd., Tokyo, Japan was used to obtain the images as well as to determine the quantitative analyses of magnetic grains. 5. Varian AA280FS atomic absorption spectrometer (AAS) made by Varian Inc., Palo Alto, CA, USA was used to measure the abundance of trace metals in bulk samples. 6. Agilent 700/725 ICP-OES (inductively coupled plasma atomic-optical emission spectrometry) made by Agilent Technologies, Santa Clara, CA, USA was used to measure the REE (rare earth elements) concentrations in bulk samples.
Data format	Raw
Experimental factors	River sediments were sieved (using 325 mesh-size sieve) and then dried at room temperature to produce bulk samples. These bulk samples were subjected to magnetic susceptibility, trace metals, and REE analyses. Some bulk samples were also subjected to magnetic extraction using magnetic stirrer and then analyzed for magnetic hysteresis parameters as well as SEM and XRD analyses. All measurements and analyses were conducted at room temperature.
Experimental features	Magnetic susceptibility measurement was conducted at dual frequencies (470 Hz and 4700 Hz). Measured magnetic hysteresis parameters are *B*_*c*_ (coercive force), *B*_*cr*_ (coercivity of remanence), *M*_*s*_ (saturation magnetization) and *M*_*rs*_ (magnetic saturation remanence). Measured trace metals are Fe, Mn, Cu, Zn, and Hg. Measured REE are La, Ce, Sc, Nd, Pr, and Gd.
Data source location	Rivers Alopohu, Bionga, and Talumelito in the vicinity of Lake Limboto in Gorontalo Province, Indonesia.
Data accessibility	The data are available with this article.
Related research article	Raghel Yunginger, Satria Bijaksana, Darharta Dahrin, Siti Zulaikah, Abd Hafidz, Kartika Hajar Kirana, Sudarningsih Sudarningsih, Mariyanto Mariyanto, and Silvia Jannatul Fajar, Lithogenic and Anthropogenic Components in Surface Sediments from Lake Limboto as Shown by Magnetic Mineral Characteristics, Trace Metals, and REE Geochemistry sediments, Geosciences 2018, 8, 116; https://doi.org/10.3390/geosciences8040116

**Table:** 

**Value of the data** • Data in this article can be used to identify the magnetic and geochemical contribution of each river that serve as inlet to Lake Limboto. • Within each river, the data could also differentiate the anthropogenic contribution as the samples were collected from pristine areas as well as the populated areas near the lake side. • Data sets can be used to correlate the magnetic properties, trace metals' content and REE concentrations; such correlations might be beneficial for environmental assessment to the seriously degraded Lake Limboto.

## 1 Data

**Fig. 1:**
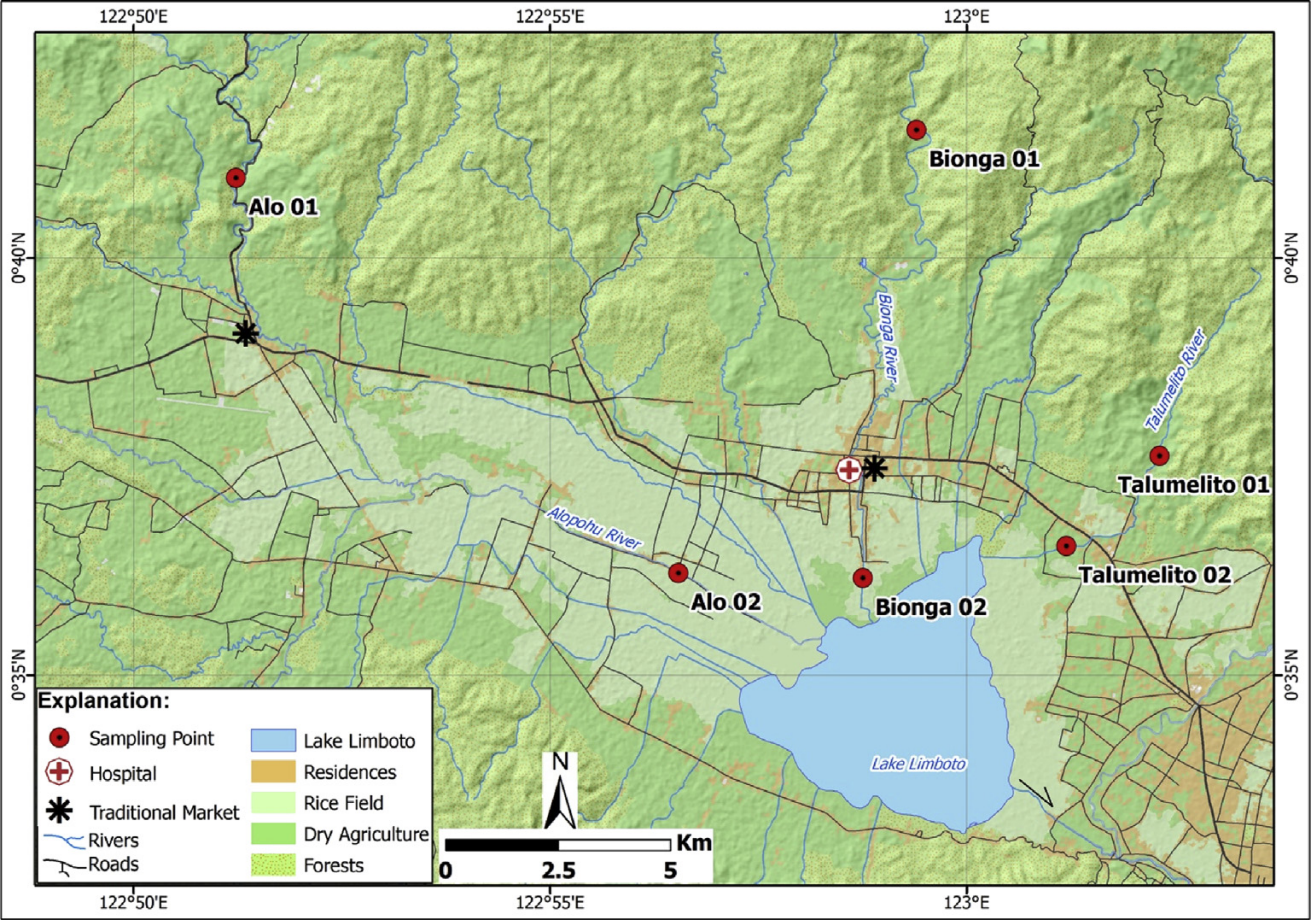
Locations of sampling points (black triangles) along Alopohu (Alo01 and Alo02), Bionga (Bionga01 and Bionga02), and Talumelito (Talumelito01 and Talumelito02) rivers that serve as inlets to Lake Limboto. Shown also traditional markets, hospital and residential areas around Lake Limboto.

Fig. 1 shows the research area that include sampling points in the three rivers (Alopohu, Bionga, and Talumelito) around Lake Limboto. Samples were obtained from two different locations along each river. The first locations are the upstream and pristine parts while the second locations are near the lake side. For each river, these two locations are separated by residential areas, markets, and even hospital. Samples are denoted as Alu01 and Alu02 (for those from Alopohu), Bionga01 and Bionga02 (for those from Bionga), and Talumelito01 and Talumelito02 (for those from Talumelito). The mineral characteristics, trace metals, REE geochemistry of surface sediments in Lake Limboto has been reported elsewhere [1]. The properties have been widely used, among others, to distinguish anthropogenic components from the lithogenic components in lake [1], [2], [3], [4], [5], [6] as well in suspended river sediments [7], [8], [9], [10].

Table 1 shows the results of magnetic susceptibility measurements in terms of χ_LF_ (low frequency mass-specific magnetic susceptibility; measured at 470 Hz), χ_HF_ (high frequency mass-specific magnetic susceptibility; measured at 4700 Hz), and χ_FD%_ (frequency-dependent magnetic susceptibility; defined as 100% × (χ_LF_ – χ_HF_)/χ_LF_). In general, the mass-specific magnetic susceptibilities of these three river sediments are much higher than that of the surface sediments in Lake Limboto [1]. Except for that of Talumelito River, the sediments near the lake are more magnetic than that from upstream locations implying that the rivers bring more magnetic anthropogenic components to the lake.

**Table 1:** Results of magnetic susceptibility measurement for sediments from the three rivers. See text for the explanation.

River	Location	Sample ID	χ_LF_ (10^−8^ m^3^/kg)	χ_HF_ (10^−8^ m^3^/kg)	χ_FD%_ (%)
Alopohu	upstream	Alo01	133.9	128.8	3.83
	lake side	Alo02	205.8	199.7	2.94
Bionga	upstream	Bionga01	162.2	158.6	2.25
	lake side	Bionga02	411.9	403.7	1.98
Talumelito	upstream	Talumelito01	211.4	202.9	4.04
	lake side	Talumelito02	168.2	159.6	5.11

**Fig. 2:**
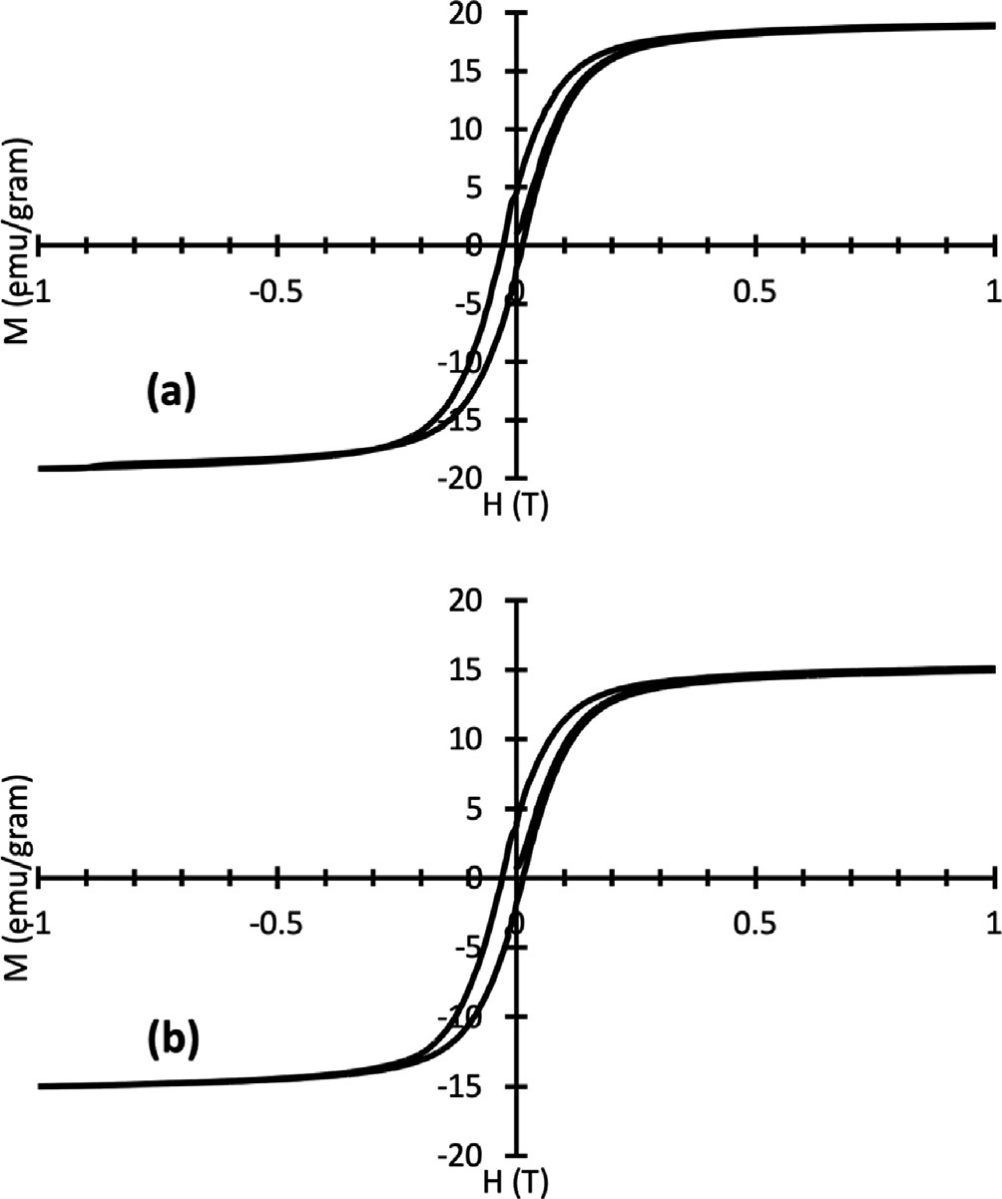
Typical magnetic hysteresis curves of extracted represented by that of Bionga01 (a) and Bionga02 (b).

**Fig. 3:**
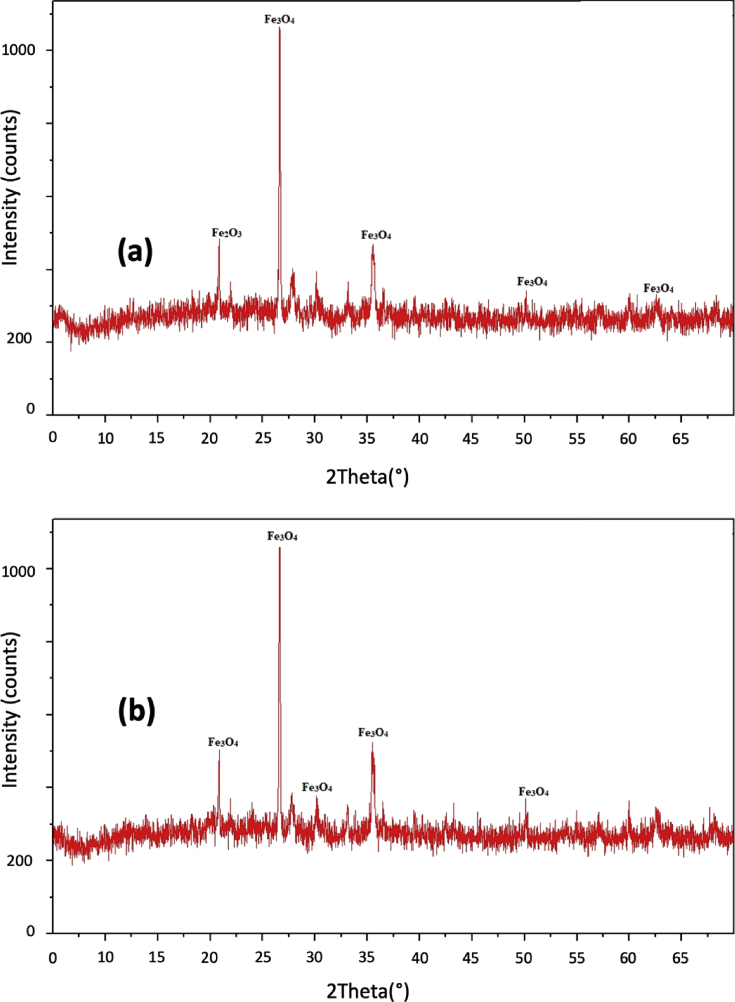
Typical X-Ray diffractograms of extracted grains represented by that of Bionga01 (a) and Bionga02 (b). The predominant mineral in the extracted grains is magnetite.

Fig. 2 shows the typical magnetic hysteresis curves for the extracted magnetic grains represented that of Bionga01 and Bionga02. The curves in Fig. 2 show that the magnetization M saturates in the field H of less than 0.3T inferring the presence of magnetite (Fe_3_O_4_) as the predominant magnetic mineral. Moreover, the presence of magnetite (Fe_3_O_4_) in the extracted grains is verified by the X-Rays diffractograms shown in Fig. 3 for that of Bionga01 and Bionga02. Meanwhile, Table 2 shows the measured magnetic hysteresis parameters, i.e., *H*_*c*_ (coercive force), *H*_*cr*_ (coercivity of remanence), *M*_*s*_ (saturation magnetization) and *M*_*rs*_ (magnetic saturation remanence) for all samples.

**Table 2:** Shows the measured magnetic hysteresis parameters for sediments from the three rivers. See text for the explanation.

River	Location	Sample ID	*H*_*c*_ (mT)	*H*_*cr*_ (mT)	*M*_*s*_ (emu/g)	*M*_*rs*_ (emu/g)	*H*_*cr*_/*H*_*c*_	*M*_*rs*_/*M*_*s*_
Alopohu	upstream	Alo01	15.8	17.3	16.7	3.5	1.09	0.21
	lake side	Alo02	5.0	19.8	24.2	5.3	3.95	0.22
Bionga	upstream	Bionga01	7.7	19.0	18.6	3.8	2.47	0.20
	lake side	Bionga02	4.3	18.4	40.4	7.0	4.29	0.17
Talumelito	upstream	Talumelito01	10.5	17.2	33.0	6.0	1.64	0.18
	lake side	Talumelito02	16.5	21.6	14.9	3.2	1.31	0.22

**Fig. 4:**
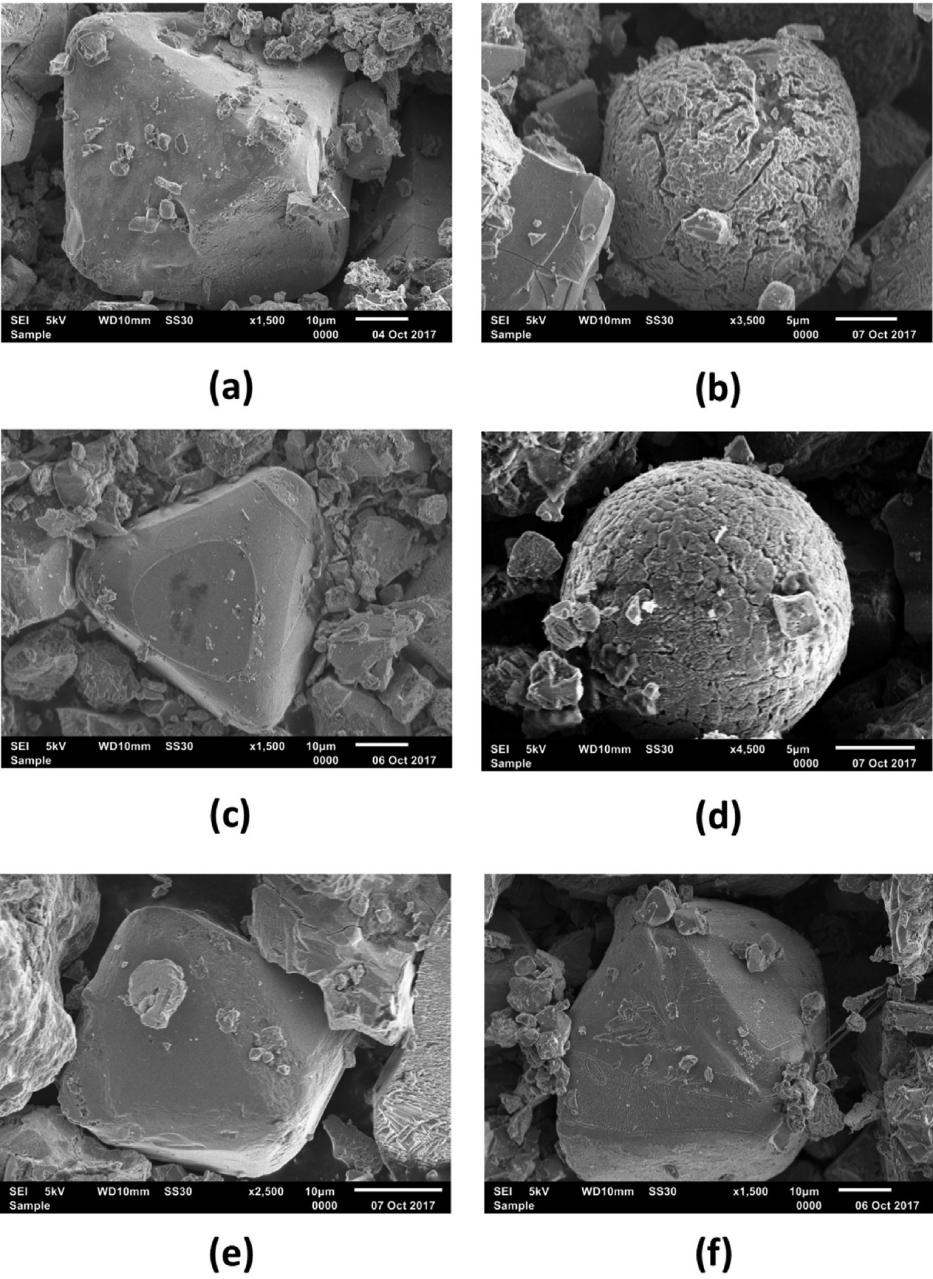
Morphologies of extracted grains from Alu01 (a), Alu02 (b), Bionga01 (c), Bionga02 (d), Talumelito01(e) and Talumelito02 (f). See text for further explanation.

The morphologies of the extracted grains are shown in SEM images in Fig. 4. The grains from upstream area are typical of natural magnetite grains, but the grains from lake side areas (Alu02 and Bionga02) are typical for anthropogenic magnetite, including framboid magnetite caused by high temperature burning. The results of EDX (energy-dispersive X-rays) analyses on grains shown in Fig. 4 are listed in Table 3.

**Table 3:** Results of EDX analyses on extracted magnetic grains shown in Fig. 4.

Element	Alopohu		Bionga		Talumelito	
	Alo01	Alo02	Bionga01 Mass%	Bionga02	Talumelito01	Talumelito02
C	5.03	5.69	8.47	8.65	7.92	8.26
N	0.60		0.04	0.78		
O	41.72	48.95	51.68	47.40	42.16	43.94
F	4.75	6.99	8.42	7.07	4.79	6.88
Na			0.14	0.03	0.12	0.20
Mg	2.24					
Al	2.41	2.38	2.31	0.76	4.11	2.21
Si	0.41	0.22	0.25	0.34	0.18	0.29
P					0.13	0.02
S		0.07				0.02
Cl	0.07		0.04			
K	0.09					
Ca	0.01					
Ti	6.98	5.09	3.17	4.96	4.29	3.69
Mn					0.08	0.07
Fe	35.71	30.15	25.36	29.90	36.14	33.85
Ni			0.12	0.10		0.19
Cu		0.45			0.09	0.16
Zn						0.22
Total	100.00	100.00	100.00	100.00	100.00	100.00

Table 4 shows the concentration of trace metals (Fe, Mn, Cu, Zn, and Hg) in the sediments from the three rivers. Compared to that of surface sediments of Lake Limboto [1], the concentrations of trace metals in river sediments are much higher. For instance, the average Fe content in surface sediment samples from Lake Limboto is only 100 ppm for residential area and is only 115 ppm for non-residential area [1].

**Table 4:** Concentration of trace metals for the sediments from the three rivers.

River	Location	Sample ID	Fe (%)	Mn (ppm)	Cu (ppm)	Zn (ppm)	Hg (ppm)
Alopohu	upstream	Alo01	5.6	1543.7	67.0	95.7	47.2
	lake side	Alo02	5.3	1509.0	50.7	104.7	27.8
Bionga	upstream	Bionga01	6.6	1428.0	78.7	99.0	66.7
	lake side	Bionga02	5.8	1783.0	72.0	150.0	44.4
Talumelito	upstream	Talumelito1	5.0	1277.0	61.7	96.0	28.9
	lake side	Talumelito2	5.6	1264.3	57.0	97.3	36.1

Table 5 shows the concentration of and of REE (La, Ce, Sc, Nd, Pr, and Gd) in the sediments from the three rivers. Compared to that of surface sediments from Lake Limboto [1], the concentrations of REE in river sediments are only slightly higher. For instance, the average Nd content in surface sediment samples from Lake Limboto is 22.50 ppm for residential area and is only 29.50 ppm for non-residential area [1]. Compared to that of sediments from Linggi River in Malaysia, the concentrations of REE in this study is about the same level for Nd, and Pr but much lower for La, Ce, and Gd [11].

**Table 5:** Concentration of REE for the sediments from the three rivers.

River	Location	Sample ID	La (ppm)	Ce (ppm)	Sc (ppm)	Nd (ppm)	Pr (ppm)	Gd (ppm)
Alopohu	upstream	Alo01	10.1	27.2	19.8	38.9	6.5	1.4
	lake side	Alo02	12.9	26.8	19.4	40.4	9.8	3.2
Bionga	upstream	Bionga01	10.2	29.6	17.3	48.5	4.8	0.6
	lake side	Bionga02	11.9	27.8	18.2	43.2	9.7	16.3
Talumelito	upstream	Talumelito1	16.6	32.3	13.9	36.1	7.2	2.2
	lake side	Talumelito2	16.3	33.9	14.3	39.7	8.8	2.8

## 2 Experimental design, materials, and methods

Sampling of sedimentary samples was conducted in six locations (see Table 6 for the coordinates of the sampling points). Samples were sieved were sieved (using 325 mesh-size sieve) and then dried at room temperature to produce bulk samples. These bulk samples were subjected to magnetic susceptibility, trace metals, and REE analyses. Magnetic susceptibility measurement was carried out using a Bartington MS2 magnetic susceptibility meter with a dual-frequencies (470 Hz and 4700 Hz) MS2B sensor (Bartington Instrument Ltd., Oxford, UK). Analyses of trace metals’ concentrations were carried out by AAS (atomic absorption spectrometer) using a Varian AA280FS (Varian Inc., Palo Alto, CA, USA) while analyses of REE concentrations were by ICP-OES (inductively coupled plasma atomic-optical emission spectrometry) using an Agilent 700/725 ICP-OES (Agilent Technologies, Santa Clara, CA, USA). Some bulk samples were then subjected to magnetic extraction using magnetic stirrer [12]. The extracted magnetic grains were then analyzed for their magnetic hysteresis parameters using an Oxford Instrument 1.2H/CT/HT vibration sample magnetometer (VSM) (Oxford Instrument, Oxfordshire, UK). Later, the extracted magnetic grains were also analyzed for their mineral composition by XRD (X-Rays diffraction) analyses using Rigaku SmartLab X-Ray Diffractometer (Rigaku Corp., Tokyo, Japan). The morphologies of these grains were also studied under scanning electron microscope (SEM) using JEOL JSM-6510A scanning electron microscope (SEM) (JEOL Ltd., Tokyo, Japan) that is also equipped with EDX (energy-dispersive X-Rays) apparatus. Table 6 lists the geographic locations of sampling points.

**Table 6:** Geographic locations of sampling points.

River	Location	Sample ID	Latitude	Longitude
Alopohu	upstream	Alo01	0° 40′ 57.60″ S	122° 51′ 13.79″ E
	lake side	Alo02	0° 36′ 47.09″ S	122° 57′ 14.10″ E
Bionga	upstream	Bionga01	0° 41′ 32.20″ S	122° 59′ 23.89″ E
	lake side	Bionga02	0° 36′ 09.69″ S	122° 58′ 45.19″ E
Talumelito	upstream	Talumelito1	0° 37′ 37.70″ S	123° 02′ 18.80″ E
	lake side	Talumelito2	0° 36′ 32.60″ S	123° 01′ 11.70″ E
